# Hepatic Stellate Cell Coculture Enables Sorafenib Resistance in Huh7 Cells through HGF/c-Met/Akt and Jak2/Stat3 Pathways

**DOI:** 10.1155/2014/764981

**Published:** 2014-06-25

**Authors:** Weibo Chen, Junhua Wu, Hua Shi, Zhongxia Wang, Guang Zhang, Yin Cao, Chunping Jiang, Yitao Ding

**Affiliations:** ^1^Department of Hepatobiliary Surgery, Affiliated Drum Tower Hospital of Nanjing University Medical School, 321 Zhongshan Road, Nanjing, Jiangsu 210008, China; ^2^Jiangsu Province's Key Medical Center for Hepatobiliary Surgery, 321 Zhongshan Road, Nanjing, Jiangsu 210008, China; ^3^School of Medicine, Nanjing University, Nanjing, Jiangsu 210093, China; ^4^Department of Hepatobiliary Surgery, Drum Tower Clinical College of Nanjing Medical University, Nanjing, Jiangsu 210008, China

## Abstract

*Purpose*. Tumor microenvironment confers drug resistance to kinase inhibitors by increasing RKT ligand levels that result in the activation of cell-survival signaling including PI3K and MAPK signals. We assessed whether HSC-LX2 coculture conferred sorafenib resistance in Huh7 and revealed the mechanism underlying the drug resistance. *Experimental Design*. The effect of LX2 on sorafenib resistance was determined by coculture system with Huh7 cells. The rescue function of LX2 supernatants was assessed by MTT assay and fluorescence microscopy. The underlying mechanism was tested by administration of pathway inhibitors and manifested by Western blotting. *Results*. LX2 coculture significantly induced sorafenib resistance in Huh7 by activating p-Akt that led to reactivation of p-ERK. LX2 secreted HGF into the culture medium that triggered drug resistance, and exogenous HGF could also induce sorafenib resistance. The inhibition of p-Akt blocked sorafenib resistance caused by LX2 coculture. Increased phosphorylation of Jak2 and Stat3 was also detected in LX2 cocultured Huh7 cells. The Jak inhibitor tofacitinib reversed sorafenib resistance by blocking Jak2 and Stat3 activation. The combined administration of sorafenib and p-Stat3 inhibitor S3I-201 augmented induced apoptosis even in the presence of sorafenib resistance. *Conclusions*. HSC-LX2 coculture induced sorafenib resistance in Huh7 through multiple pathways: HGF/c-Met/Akt pathway and Jak2/Stat3 pathway. A combined administration of sorafenib and S3I-201 was able to augment sorafenib-induced apoptosis even in the presence of LX2 coculture.

## 1. Introduction

Hepatocellular carcinoma (HCC) is currently the fifth most common solid tumor worldwide, causing almost 700,000 deaths every year, making it the third leading cause of cancer-related death [1]. Surgical resection remains the major therapy for HCC patients of early stage. However, HCC is often diagnosed at an advanced stage, when most potentially curative therapies are of limited functions. Recently, much attention has been paid to the discovery of novel therapeutics for HCC patients of advanced stage. Despite all the progress, the prognosis of HCC is still not satisfying, with the total 5-year survival rate being at 12% [2, 3].

Sorafenib is a multitargeted tyrosine kinase inhibitor that blocks the Ser/Thr kinase Raf and several receptor tyrosine kinases including vascular endothelial growth factor receptor- (VEGFR-) 2 and -3 and platelet-derived growth factor receptor- (PDGFR-) *β* to inhibit tumor cell proliferation and tumor angiogenesis. Sorafenib has been approved by the Food and Drug Administration (FDA) for the treatment of unresectable HCC since late 2007 based on the results from a phase III clinical trial that demonstrated that sorafenib improves overall survival and is safe for patients with advanced HCC.

Although sorafenib is currently the only approved pharmacological therapy for HCC, the overall tumor response rates are unfortunately low. The antitumor efficiency of sorafenib correlates with the inhibition of MAPK signaling and the reactivation of ERK has been discovered to cause sorafenib resistance. It has been reported that cancer cells typically express multiple receptor tyrosine kinases (RTKs) that mediate the upregulation of cell-survival effectors, generally, phosphatidylinositol-3-OH kinase (PI3K) and mitogen-activated protein kinase (MAPK) [4]. The increase of RTK-ligands, either from autocrine or paracrine, was able to confer resistance to kinase inhibitors [5]. Lately, tumor microenvironment was found to confer innate resistance to targeted agents. By coculture system, Ravid et al. found that stromal cell could secret hepatocyte growth factor (HGF) that resulted in the resistance to RAF inhibitors in melanoma and breast cancer cells [6].

In this study, by using coculture system we aimed to discover whether the stromal cells in HCC microenvironment could confer sorafenib resistance and to reveal the underlying mechanism beneath the drug resistance.

## 2. Materials and Methods

### 2.1. Cells and Reagents

The human HCC cell lines Huh7 and PLC/PRF/5 were purchased from Cell Bank of Chinese Academy of Sciences (Shanghai, China). Human hepatic stellate cell (HSC) line HSC-LX2 was purchased from Cell Bank of Xiangya Central Experiment Laboratory of Central South University (Changsha, China). MHCC-97H and MHCC-97L cell lines were obtained from the Liver Cancer Institute, Fudan University (Shanghai, China). Huh7 and HSC-LX2 were supported with Dulbecco's Modified Eagle Medium (DMEM, WISENT, CA) containing 10% fetal bovine serum (FBS) (ExCell Bio, China). Both cells were incubated in 5% CO_2_ at 37°C. Sorafenib tosylate, Met inhibitor crizotinib (PF-02341066), the p-Akt inhibitor MK-2206 2HCl, the Janus kinase (Jak) inhibitor tofacitinib (CP-690550), and the signal transducer and activator of transcription 3 (Stat3) inhibitor S3I-201 were purchased from Selleck (Selleck Chemicals, China). MTT (3-(4,5-dimethylthiazol) 2, 5-diphenyltetrazolium) was purchased from Sigma Aldrich (St. Louis, MO). The antibodies for glyceraldehyde-3-phosphate dehydrogenase (GAPDH), p-Met, p-Akt, Akt, p-ERK, ERK, p-Stat3, Stat3, caspase-9, caspase-3, poly (ADP-ribose) polymerase (PARP), and p-Jak2 were obtained from Cell Signaling Technology (Beverly, MA). The antibodies for Bcl-2, Mcl-1, and Bax were purchased from Enogene (Nanjing, China). The antibodies for VEGFR-3 and c-kit were obtained from Bioworld Technology Inc. (Bioworld, USA). The antibody for PDGFR-*β* was purchased from Santa Cruz Biotechnology Inc. (Santa Cruz, USA). ELISA kit for human HGF detection was purchased from ExCell Bio (Shanghai, China).

### 2.2. Cell Coculture Model

For LX2 cell coculture, 6-well 0.4 *μ*m Millicell hanging cell culture inserts (Switzerland) were used. Generally, 5 × 10^5^ Huh7 cells were placed in 6-well plate while 1 × 10^5^ LX2 cells were placed in hanging cell inserts. For coculture in 96-well plates, the culture supernatants of LX2 after incubating for 48 h were collected and centrifuged at 5000 rpm before it was distributed for Huh7 culture for 72 h.

### 2.3. Cell Viability Assay

Cell viability was monitored using MTT assay. Generally, 5 × 10^3^ cells were allowed to grow in 96-well plates. After incubation with sorafenib for 48 h, 20 *μ*L MTT solution (0.5%) was added to the medium for further incubation for 4 h. 150 *μ*L DMSO was added to every well to dissolve the insoluble formazan product after removing the medium. The absorbance of the colored solution was measured at 570 nm with a spectrophotometer. All experiments were performed in triplicates.

### 2.4. Western Blotting

Cell lysates prepared after 48 h of administration of sorafenib were immunoblotted following published protocol [7]. The signal was developed with ECL (Millipore, Switzerland) after incubation with appropriate second antibody.

### 2.5. ELISA Assay

HGF concentration in LX2 supernatants was detected by human HGF ELISA kit (ExCell, China) following the manufacturer's instruction. Briefly, after cultivating HSC-LX2 for 48 h, the medium was collected and centrifuged at 5000 rpm for 5 min. Total medium with 10% FBS was set as control.

### 2.6. Real-Time PCR

ETS-1 expression level was detected by real-time PCR. Briefly, total RNA was extracted by using TRIzol reagent (TaKaRa, Japan) and reverse transcription was performed using Primescript RT master mix (TaKaRa, Japan). The cDNA was subjected to quantitative real-time PCR using the SYBR Green PCR Kit (TaKaRa, Japan) and the assay was performed on ABI PRISM 7300 Sequence Detector. *Β*-actin was used as the internal control. The relative expression level was calculated by 2^−ΔΔCt^ method. The primer sequences for ETS-1 and *β*-actin were as follows: ETS-1 forward: 5′-GTTAATGGAGTCAACCCAGCCTATCC-3′; ETS-1 reverse: 5′-GGGGTGACGACTTCTTGTTTGATAGC-3′; *β*-actin forward: 5′-CAGGCACCAGGGCGTGATGG-3′; *β*-actin reverse: 5′-CTGTAGCCGCGCTCGGTGAG-3′.

### 2.7. DAPI Staining

Cell apoptosis was detected by 4′,6-diamidino2-phenylindole (DAPI) staining which allowed identification of apoptotic nuclear changes. Briefly, cells were washed with PBS and fixed with 4% paraformaldehyde at room temperature for 30 min. After being washed with PBS, cells were stained with 10 *μ*g/mL DAPI for 5 min. Cells on slides were subjected to fluorescence microscopic examination.

### 2.8. Statistical Analysis

All the data were expressed as mean ± SD from three individual experiments. Differences between groups were determined by using the Student's *t*-test and two-way ANOVA with Bonferroni correction. *P* < 0.05 was considered significant.

## 3. Results

### 3.1. HSC-LX2 Coculture Induced Sorafenib Resistance in Huh7

**Figure 1:**
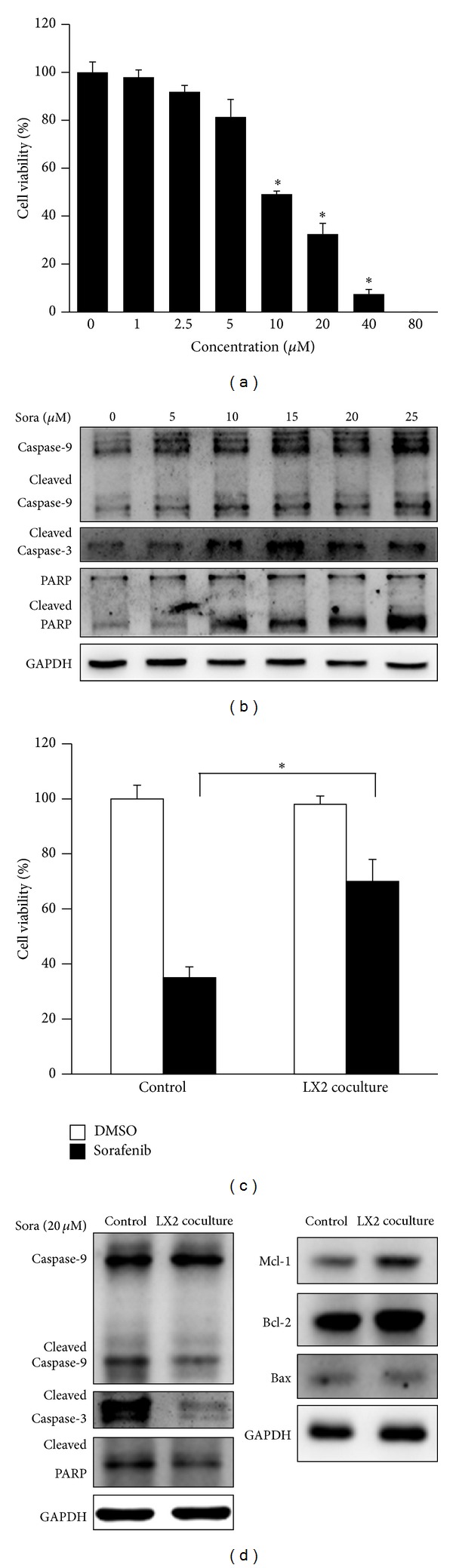
HSC-LX2 coculture induced sorafenib resistance in Huh7. (a) The inhibitory effect of sorafenib on Huh7 cells. After 48 h of sorafenib exposure at different concentrations, cell viability was assessed by MTT assay. Sorafenib could impair Huh7 cell viability at the concentration equal to and more than 10 *μ*M. (b) Sorafenib-induced apoptosis in Huh7. After 48 h of sorafenib exposure at different concentrations, cell apoptosis was assessed by Western blotting. (c) HSC-LX2 coculture attenuated sorafenib-induced cell suppression. Huh7 cells were cocultured with LX2 for 48 h before the administration of sorafenib for another 48 h. Cell viability was assessed by MTT assay which showed attenuated cell death in cocultured group. (d) HSC-LX2 coculture attenuated sorafenib-induced apoptosis by increasing antiapoptotic proteins. Cell lysates were subjected to Western blotting after sorafenib administration for 48 h. HSC-LX2 coculture decreased cleavage of caspases and PARP (left panel). HSC-LX2 coculture increased expression levels of antiapoptotic proteins Mcl-1 and Bcl-2 (right panel) (*P < 0.05; sora: sorafenib).

To investigate whether HSC-LX2 coculture induced sorafenib resistance in HCC cell lines, we cocultured several HCC cell lines with LX2 supernatants for 48 h and then assigned the cells to administration of sorafenib. We found that LX2 coculture impaired cell viability of MHCC-97H, MHCC-97L, and PLC/PRF/5, but not Huh7, so we selected Huh7 cells for further investigations (data not shown). The impaired cell viability might be the results of LX2 coculture induced epithelial-mesenchymal transition (EMT) [8]. We first confirmed that sorafenib suppressed proliferation and induced apoptosis in Huh7 as reported [9]. The effect of sorafenib on cell proliferation was measured by MTT assay. Sorafenib inhibited cell viability dose-dependently in Huh7, with the inhibition rate being at about 50% for 10 *μ*M and about 60% for 20 *μ*M (Figure 1(a)). The IC50 value of sorafenib for Huh7 was 11.25 *μ*M. Furthermore, sorafenib administration induced elevated protein level of cleaved caspase-9, cleaved caspase-3, and cleaved PARP dose-dependently (Figure 1(b)).

We utilized MTT assay to verify the induced sorafenib resistance in Huh7. LX2 coculture for 48 h significantly attenuated sorafenib-induced cell viability suppression (Figure 1(c)). What was more, LX2 coculture also reversed the cleavage of caspase family, as the cleaved form of caspase-9, caspase-3, and PARP decreased in LX2 cocultured Huh7 after sorafenib administration. We also detected an increased level of the antiapoptotic proteins Mcl-1 and Bcl-2; however, no obvious change was detected as to proapoptotic protein Bax (Figure 1(d)).

### 3.2. Elevated HGF in LX2 Coculture Supernatant Contributed to Sorafenib Resistance

**Figure 2:**
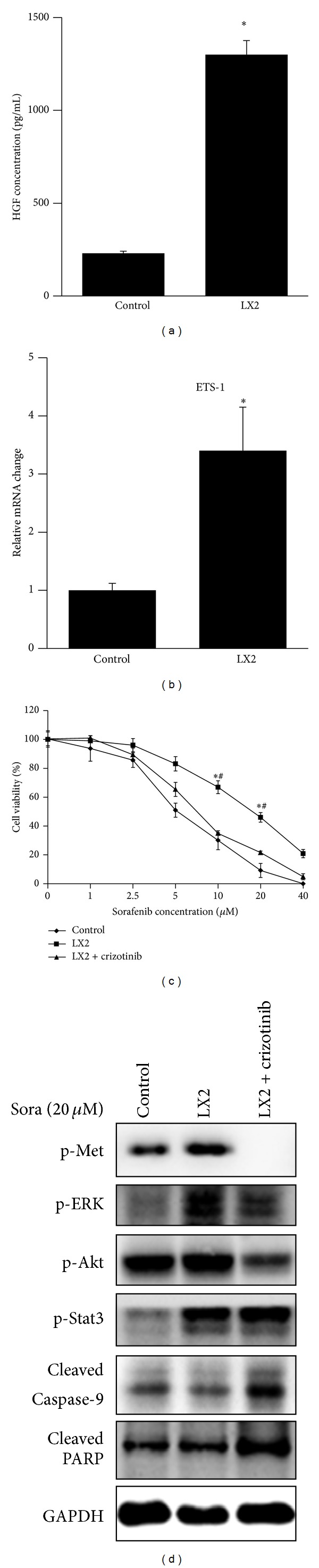
Elevated HGF in LX2 coculture supernatant contributed to sorafenib resistance. (a) HGF increased in LX2 supernatants. HGF concentration in LX2 supernatants was determined by ELISA kits. HGF in LX2 supernatants increased more than 5 times than control medium to the concentration of almost 1300 pg/mL. (b) ETS-1 expression level in LX2 cocultured Huh7 was determined by real-time PCR. ETS-1 mRNA level increased about 3 times than control. (c) Administration of crizotinib reversed sorafenib resistance. 500 nM crizotinib was added to the coculture system for 48 h when administrating sorafenib. Cell viability was assessed by MTT assay. Crizotinib reversed coculture induced cell survival while it alone impaired no cell viability. (d) Crizotinib reversed inactivation of caspase cleavage by inhibiting p-Met, p-ERK, and p-Akt. Crizotinib inhibited phosphorylation of Met, ERK, and Akt while it did not affect Stat3 (*P < 0.05 LX2 versus control; ^#^ *P* < 0.05 LX2 versus LX2 + crizotinib. sora: sorafenib).

To find out what was the compound in LX2 supernatants that caused sorafenib resistance, we screened the concentration of HGF in LX2 supernatants which was reported to cause elotinib resistance in breast cancer [6, 10]. Interestingly, the results indicated that HGF concentration in LX2 supernatants increased more than 5 times compared to control medium (Figure 2(a)). Because the stimulated expression of c-Met in human endothelial cells by HGF was largely dependent on the induction of essential transcription factors (ETS), especially ETS-1(11), we further checked the expression level of ETS-1 in LX2 coculture Huh7, and elevated ETS-1 mRNA level was detected (Figure 2(b)). To further confirm that HGF reverted resistance to sorafenib, crizotinib (PF234106) which was a potent c-Met inhibitor was adopted for further studies. Crizotinib abrogated LX2 coculture induced sorafenib resistance by decreasing the cell viability (Figure 2(c)). Changes in the phosphorylation of Met, ERK, Akt, and Stat3 and cleavage of caspase-9 and PARP were determined by Western blotting to evaluate the effect of crizotinib on c-Met pathway and apoptosis. Crizotinib inhibited the phosphorylation of Met significantly and also inhibited p-ERK and p-AKt at the dose of 500 nM. No change of p-Stat3 was detected upon crizotinib administration. Crizotinib also increased the level of cleaved form of PARP and caspase-9 which turned to be the reversal of sorafenib resistance (Figure 2(d)).

### 3.3. Exogenous HGF Could Also Trigger Sorafenib Resistance in Huh7

**Figure 3:**
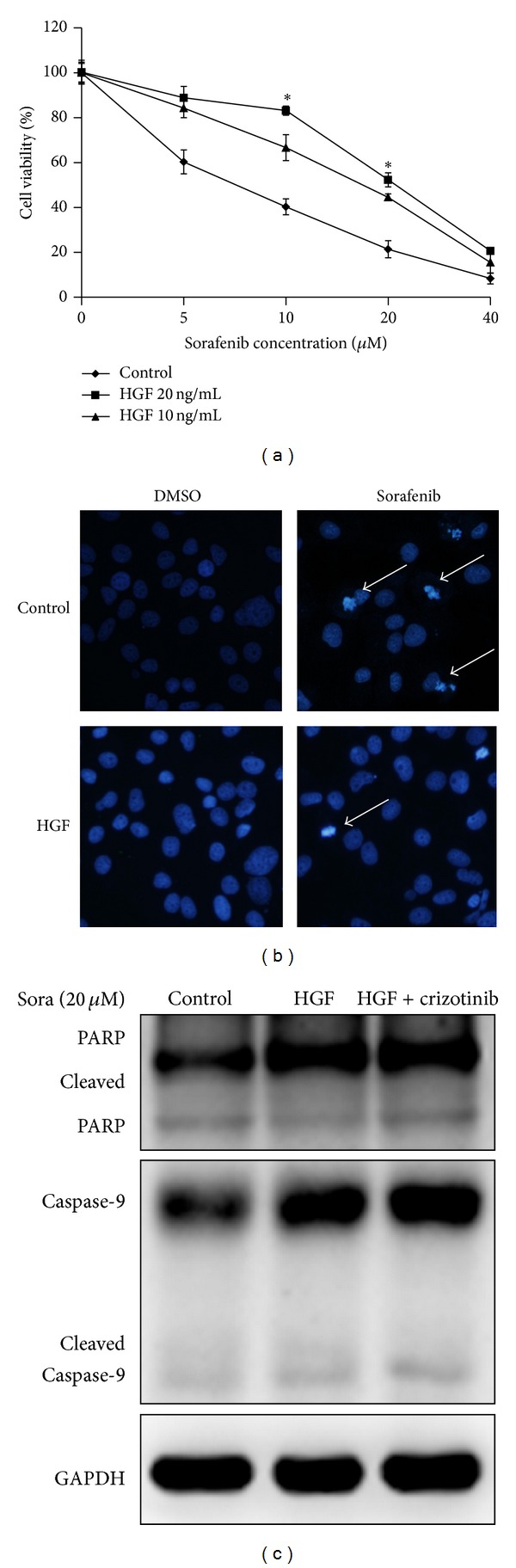
Exogenous HGF could also trigger sorafenib resistance in Huh7. (a) HGF of indicated concentration was administrated 2 h before sorafenib administration. HGF of 20 ng/mL rescued Huh7 from cell death. (b) DAPI staining was adopted for the manifestation of cell death. HGF of 20 ng/mL decreased sorafenib-induced cell death. (c) Crizotinib reversed HGF-induced cell survival. Crizotinib activated the cleavage of PARP and caspase-9 (*P < 0.05 HGF 10 ng/mL versus control; sora: sorafenib).

Having established that it was HGF secreted by LX2 that was responsible for sorafenib resistance, we sought to determine whether exogenous HGF could also trigger sorafenib resistance. Therefore Huh7 cells were pretreated with HGF at different concentrations (10 ng/mL and 20 ng/mL) for 2 h before the treatment of 20 *μ*M sorafenib for 48 h. The cell viability was subsequently assessed using MTT assay. The results showed that HGF of 20 ng/mL attenuated sorafenib-induced cell death significantly (Figure 3(a)). Cell death was also assessed by DAPI staining and Western blotting which turned out to be consistent in demonstrating the rescue effect of HGF in saving Huh7 cells from sorafenib-induced cell death (Figures 3(b) and 3(c)).

The above data showed that HGF secreted by LX2 into the supernatants was responsible for LX2 coculture induced sorafenib resistance in Huh7, and the pretreatment with exogenous HGF could also induce sorafenib resistance.

### 3.4. Inhibition of p-Akt Reversed LX2 Coculture Induced Sorafenib Resistance

**Figure 4:**
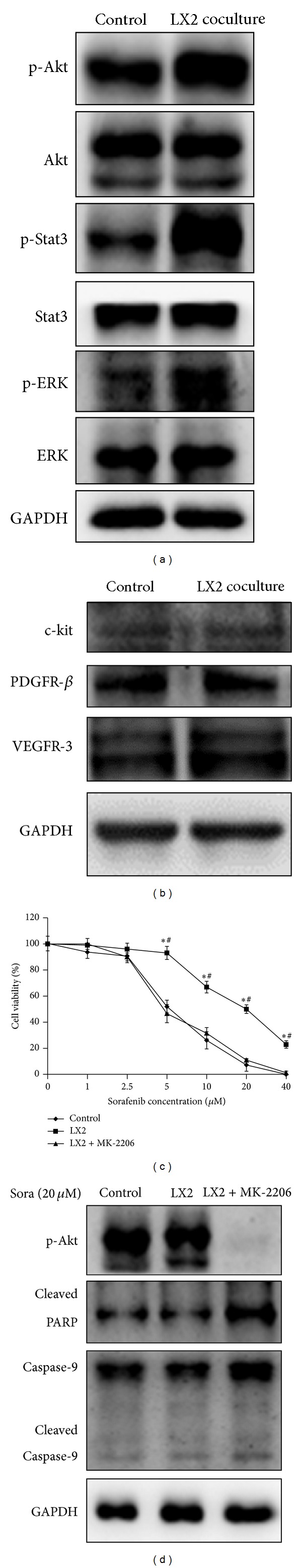
Inhibition of p-Akt reversed LX2 coculture induced sorafenib resistance. (a) Signaling pathway changed in LX2 cocultured Huh7. p-ERK, p-Akt, and p-Stat3 increased in LX2 cocultured Huh7. (b) LX2 coculture did not impact c-kit, PDGFR-*β*, and VEGFR-3 protein level. (c) Inhibition of p-Akt by MK-2206 reversed LX2 coculture induced sorafenib resistance. 10 *μ*M MK-2206 was added to the coculture system for 48 h when administrating sorafenib. Cell viability was assessed by MTT assay. MK-2206 reversed coculture induced cell survival while it alone impaired no cell viability. (d) MK-2206 reversed inactivation of caspase cleavage by inhibiting p-Akt. MK-2206 significantly inhibited p-Akt and reactivated PARP and caspase-9 cleavage (*P < 0.05 LX2 versus control; ^#^ *P* < 0.05 LX2 versus LX2 + MK-2206; sora: sorafenib).

To characterize the mechanism responsible for sorafenib resistance in Huh7, we first focused on the MAPK, AKT, and Stat3 signaling pathway, because sorafenib inhibited the phosphorylation of ERK in HCC cells and induced apoptosis [11]. The activation of PI3 K/Akt signaling pathway mediated the acquired resistance to sorafenib in HCC [9]. Sorafenib could downregulate p-Stat3 in a dose- and time-dependent manner in HCC cells, while p-Stat3 activation was discovered to be the cause of recombinant tumor necrosis factor-related apoptosis-inducing ligand (TRAIL) resistance. Therefore, the phosphorylation of ERK, Akt, and Stat3 was assessed by Western blotting. The results indicated an increased level of phosphorylation of ERK, Akt, and Stat3 in LX2 cocultured Huh7 (Figure 4(a)). However, we did not find the change in the protein level of RTKs like c-kit, PDGFR-*β*, and VEGFR-3 (Figure 4(b)).

We hypothesized that the reactivation of ERK by LX2 coculture was due to the activation of p-Akt, and p-Stat3 played a role in mediating the drug resistance. Thus, we validated the role of Akt in the effects of sorafenib resistance by blocking Akt phosphorylation with MK-2206, an Akt inhibitor. Our data showed that the presence of MK-2206 was sufficient to reverse LX2 coculture induced sorafenib resistance at the dose of 10 *μ*M (Figure 4(c)). The combination of sorafenib and MK-2206 significantly increased the percentage of apoptotic cells in LX2 cocultured Huh7 while MK-2206 alone affected no cell viability at the concentration of 10 *μ*M. In addition, downregulation of p-Akt sensitized Huh7 cells to sorafenib-induced apoptosis, as showed by the activation of caspase-9 and PARP cleavage (Figure 4(d)).

### 3.5. Inhibition of Jak2/Stat3 Reserved LX2 Coculture Induced Sorafenib Resistance

**Figure 5:**
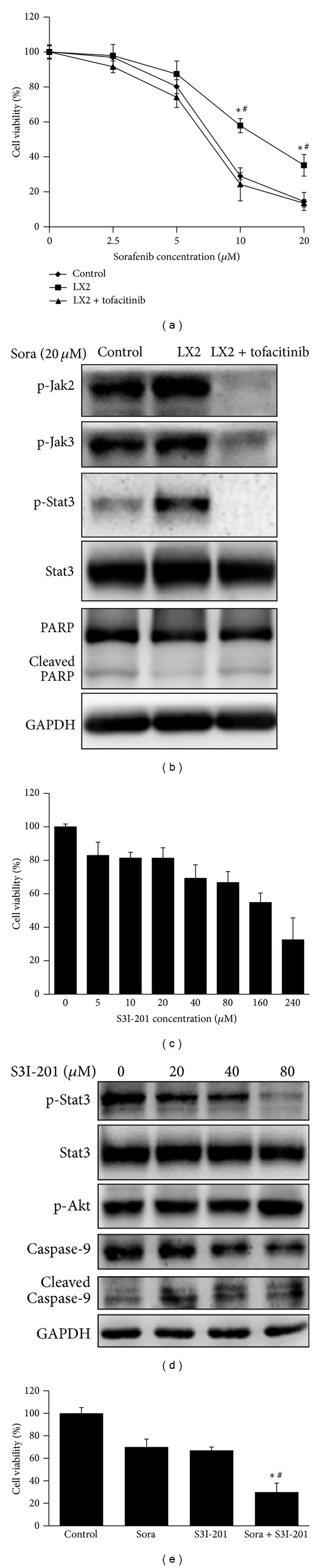
Inhibition of p-Stat3 reserved LX2 coculture induced sorafenib resistance. (a) Jak inhibitor tofacitinib reversed LX2 coculture induced sorafenib resistance. 0.25 *μ*M tofacitinib was added to the coculture system for 48 h when administrating sorafenib. Cell viability was assessed by MTT assay. Tofacitinib reversed coculture induced cell survival while it alone impaired no cell viability. (b) Activation of Jak2/Stat3 induced sorafenib resistance whose inhibition activated sorafenib-induced apoptosis. (c) Stat3 inhibitor S3I-201 inhibited cell viability dose-dependently. (d) S3I-201 inhibited phosphorylation of Stat3 and induced apoptosis dose-dependently. (e) Combined administration of sorafenib (20 *μ*M) and S3I-201 (80 *μ*M) augmented cell apoptosis in the presence of LX2 coculture (*P < 0.05 sora + S3I-201 versus sora; ^#^sora + S3I-201 versus S3I-201; sora: sorafenib).

Jak inhibitor tofacitinib was also adopted in our research which showed that tofacitinib at the concentration of 0.25 *μ*M reversed sorafenib resistance by decreasing the cell viability while it alone did not impact cell viability (Figure 5(a)). Jak2 but not Jak3 activation was responsible for Stat3 activation. The inhibition of Jak2 blocked Stat3 activation and induced the activation of PARP cleavage (Figure 5(b)).

We then further investigate whether the direct inhibition of p-Stat3 could reverse sorafenib resistance. We used S3I-201 to inhibit p-Stat3 activity. S3I-201 was a direct Stat3 inhibitor that blocks Stat3 dimerization and DNA-binding and transcriptional activities. The therapeutic effect of S3I-201 in xenografts of HCC cell line Huh7 was that it inhibited Stat3 tyrosine phosphorylation and tumor growth at a dose of 5 mg/kg given every other day [12]. Based on the above research, we first tested the inhibitory effect of S3I-201 on Huh7. We found that S3I-201 inhibited Huh7 cell viability dose-dependently, and it inhibited p-Stat3 activation also in a dose-dependent manner. At the optimized dose of 80 *μ*M, S3I-201 could significantly inhibit Stat3 phosphorylation and impair Huh7 cell viability. Treatment of S3I-201 alone could activate the cleavage of caspase-9 (Figures 5(c) and 5(d)). As the inhibition of p-Stat3 alone could induce apoptosis in Huh7, we next tried to figure out whether the combination of sorafenib and S3I-201 could promote the apoptosis in Huh7 in the presence of LX2 supernatants. To our delight, S3I-201 could induce severer cell death than sorafenib even in the presence of LX2 coculture, and the combination of sorafenib and S3I-201 augmented the induced apoptosis (Figure 5(e)).

## 4. Discussion

Sorafenib is a multitargeted tyrosine kinase inhibitor that blocks the Ser/Thr kinase Raf and several receptor tyrosine kinases including VEGFR-2 and -3 and PDGFR-*β* to inhibit tumor cell proliferation and tumor angiogenesis. It has been approved by FDA for the treatment of unresectable HCC based on the results from a phase III clinical trial that demonstrated its benefits for overall survival and the safety for patients with advanced HCC. However, sorafenib resistance is a major obstacle in improving therapeutic efficacy. Stromal cells in tumor microenvironment have been known to be capable of secreting RTK ligands to confer resistance to kinase inhibitors. In this report, we discovered that HSC-LX2, the stromal cell in HCC microenvironment, was able to confer sorafenib resistance in Huh7 cells by secreting HGF into the culture medium which could increase the phosphorylation of Met, Akt, ERK, and Stat3. Exogenous HGF was also able to trigger sorafenib resistance. LX2 coculture induced sorafenib resistance could be reversed by the administration of crizotinib, MK-2206, and tofacitinib, the potent inhibitors of Met, Akt, and Jak, respectively. In addition, we found that when combining administration of sorafenib and S3I-201, the inhibitor of Stat3 augmented induced apoptosis even in the presence of sorafenib resistance.

Tumor microenvironment has been demonstrated to confer drug resistance that leads to relapse and incurability of cancers. Soluble factors as cytokines and growth factors secreted by stromal cells are a major cause of acquired resistance [13]. Among the various factors, HGF is found to confer substantial resistance to RAF and MEK inhibition by activation of the RTK Met in melanoma and breast cancers [6]. Activation of HGF/c-Met axis elicits multiple cellular responses regulating cell survival [14]. The study by Yu et al. shows that HSC-LX2 cell secreted HGF contributes to the chemoresistance of HCC. The elevated HGF level instead of TGF-*β* activates Met in HCC cell Hep3B [8]. In our study, increased level of HGF was also detected which contributed to sorafenib resistance. The blockage of HGF/c-Met axis by crizotinib reversed drug resistance. However, there was a big gap between the HGF concentration in LX2 supernatants and the concentration we used for exogenous HGF stimulation experiments. We supposed that the different HGF exposure time might be the reason for the gap.

PI3K/Akt signaling is a vital survival and proliferative pathway involving many growth factors, cytokines, and activation of receptors. Chen et al. have reported that activation of PI3K/Akt signaling pathway mediates acquired resistance to sorafenib in HCC, and the combined administration of sorafenib and MK-2206 overcomes the resistance [9]. In our study, LX2 coculture increased the activation of p-Akt and thus decreased the activation of caspase and PARP cleavage and increased the antiapoptotic protein Mcl-1 and Bcl2 levels. While under the administration of crizotinib and MK-2206, when both inhibitors inhibit the phosphorylation of Akt indirectly or directly, the rescue effect was reversed. As sorafenib blocks the RAF/MEK/ERK pathway to inhibit tumor angiogenesis and induce apoptosis in HCC cells, the reactivation of ERK signaling confers acquired resistance to BRAF and MEK inhibitors [11, 15]. As shown in Figures 2(d) and 4(a), p-ERK increased in LX2 cocultured Huh7 cells and no c-kit, PDGFR-*β*, or VEGFR-3 protein level increase was detected (Figure 4(b)); we assumed that ERK activation might be the direct reason for induced resistance. Because the inhibition of p-Met by crizotinib can partially block the activation of p-ERK, crizotinib reverses sorafenib resistance (Figure 2(d)). So in LX2 cocultured induced sorafenib resistance in Huh7, LX2 secreted HGF into the tumor microenvironment which activated HGF/c-Met axis and thus activates p-Akt and then reactivated p-ERK somehow which finally led to the increased level of antiapoptotic protein and decreased caspase cleavage.

Because crizotinib did not affect Stat3 activation as shown in Figure 2(d), we assumed that c-Met activation was not responsible for Stat3 activation. We observed Jak2 activation but not Jak3 activation in LX2 cocultured Huh7, and Jak inhibitor tofacitinib reversed sorafenib resistance by inhibiting Jak2 and Stat3 activation. We also found that the direct inhibition of p-Stat3 induced apoptosis. The inhibition of p-Met affected no p-Stat3 activation and inhibition of p-Stat3 affected no p-Akt, either. There seemed no cross-talk between p-Akt and p-Stat3 signaling pathway. Stat3 is associated with cell proliferation and survival and it is crucial in the prognosis of many cancers and a potential target for anticancer therapy [16, 17]. It has been reported that Stat3 is a major kinase-independent target of sorafenib in HCC and sorafenib downregulates p-Stat3 and reduces the expression levels of Stat3-related proteins as Mcl-1, survivin, and cyclin D1 by upregulating SHP-1 activity [18–21]. The Stat3 inhibitor, S3I-201, has been demonstrated to inhibit Stat3 dimerization and DNA-binding activity as well as inhibit cell proliferation and tumor growth [22, 23]. Stat3 inhibition combined with targeted therapy has been proved to significantly suppress cancer cell growth in pancreatic cancers [24]. In our study, the combined therapy of S3I-201 and sorafenib synergistically suppressed Huh7 cell growth and induced apoptosis in the presence of sorafenib resistance.

In summary, HSC-LX2 coculture induced sorafenib resistance in Huh7 through two independent pathways: HGF/c-Met/Akt pathway and Jak2/Stat3 pathway. A combined administration of sorafenib and S3I-201 was able to augment sorafenib-induced apoptosis even in the presence of sorafenib resistance.
